# RNY1 Is Heterogeneously Partitioned in Inflamed Airway Fluid and Modulates Pro‐Inflammatory Macrophage Transcriptional Programming

**DOI:** 10.1002/jex2.70162

**Published:** 2026-07-03

**Authors:** Cherie E. Saffold, Antiana C. Richardson, Heather H. Pua

**Affiliations:** ^1^ Department of Pathology, Microbiology, and Immunology Vanderbilt University Medical Center Nashville Tennessee USA; ^2^ Vanderbilt Institute for Infection, Immunology, and Inflammation Vanderbilt University Medical Center Nashville Tennessee USA; ^3^ Vanderbilt Center for Immunobiology Vanderbilt University Medical Center Nashville Tennessee USA; ^4^ Vanderbilt Center for Extracellular Vesicle Research Vanderbilt University Nashville Tennessee USA

**Keywords:** bronchoalveolar lavage fluid, extracellular RNA, extracellular vesicle, lung inflammation, macrophage, RNY1, YRNA

## Abstract

YRNAs are small noncoding RNAs that are abundant in the extracellular space. Prior research has shown that YRNAs are present in most biofluids and that their levels fluctuate during disease. Yet our understanding of YRNA dynamics in the context of biofluid extracellular particles during disease processes is limited. In this study, we found that RNY1, one of two murine YRNAs, increases in airway fluid during allergen‐induced lung inflammation and correlates with neutrophil infiltration. Using RNase sensitivity assays and size exclusion chromatography, we determined that airway fluid RNY1 is protected by both EVs and protein‐containing structures in distinct extracellular compartments. In contrast, the other murine YRNA, RNY3, is only enriched in BALF EVs, suggesting that RNY1 is uniquely partitioned in heterogeneous extracellular particles. Both EV‐enriched and protein‐enriched RNY1‐containing extracellular compartments were able to program robust pro‐inflammatory transcriptional responses in target macrophages. RNY1 contributed to this programming, as macrophages treated with a protein‐enriched compartment isolated from *RNY1^−/−^* mice demonstrated lower induction of interferon‐associated genes. Together, this study demonstrates that YRNAs are heterogeneously packaged in complex biofluids and contribute to intercellular signalling axes during inflammation.

## Graphical abstract

Key Findings Summary: Extracellular RNY1 increases in airway fluid and correlates with levels of neutrophil infiltration in the lung during allergen‐induced inflammation. This YRNA is partitioned into both extracellular vesicle (EV)‐ and protein‐enriched airway fluid fractions and promotes transcriptional reprogramming of macrophages.

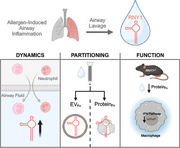

**Abbreviations**
BALF: bronchoalveolar lavage fluid
BMDM: bone marrow‐derived macrophage
EP: extracellular particle
EV: extracellular vesicle
EV_Fx_
: EV‐enriched fraction
exRNA: extracellular RNA
HDM: house dust mite
miRNA: microRNA
NVEP: non‐vesicular extracellular particle
OVA: ovalbumin
PK: proteinase K
Protein_Fx_
: protein‐enriched fraction
SEC: size exclusion chromatography
Tx: Triton X‐100

## 1 Introduction

Extracellular RNAs (exRNAs) are present in all biofluids and have garnered widespread interest due to their potential as diagnostic biomarkers (Godoy et al. 2018, Mori et al. 2019, Zhao et al. 2024, Quinn et al. 2015). Growing evidence also points to critical roles for exRNAs in cell‐to‐cell communication(Baglio et al. 2016, Nabet et al. 2017, Tsatsaronis et al. 2018, Cheng & Shorey 2019). However, the packaging, transport and function of individual exRNAs in vivo remains poorly understood. While many classes of RNAs are secreted into the extracellular space, exRNAs are enriched for small noncoding RNAs that are <200 nucleotides in length and play critical regulatory roles in the cell. The best studied RNAs in this class are microRNAs (miRNAs), which are selectively secreted and capable of regulating gene expression post‐transcriptionally in recipient cells (Nation et al. 2021, Momen‐Heravi et al. 2015, Bouchareychas et al. 2020, Bouchareychas et al. 2020). Yet, miRNAs comprise only a small fraction of all exRNAs (Wei et al. 2017, Murillo et al. 2019, Godoy et al. 2018). The secretion and function of other exRNA species remains a critical gap in our understanding of intercellular communication via RNA.

Among the most commonly detected exRNAs are YRNAs, which are secreted by various cells, including cancer cells, immune cells, and stem cells (Wei et al. 2017, Nolte'T Hoen et al. 2012, van Balkom et al. 2015). YRNAs are highly conserved, with YRNAs or YRNA‐orthologs identified in a wide variety of species from vertebrates to bacteria and archaea (Kowalski & Krude 2015, Perreault et al. 2007). The two murine YRNAs (RNY1 and RNY3) are broadly expressed RNA Polymerase III transcripts that exist as ∼110 nucleotide stem‐loop structures. In addition, YRNAs can be cleaved into smaller fragments, though the regulation and function of these cleavage events are not well understood (Dhahbi et al. 2013, Nechooshtan et al. 2020). Some studies have shown that YRNAs bind to protein complexes that control the degradation of intracellular RNAs (Chen et al. 2013). However, these experiments were performed in UV‐stressed bacterial cells, and our understanding of YRNA function in mammalian systems remains limited.

Like other exRNAs, YRNAs are commonly identified in biofluids, including plasma, urine and saliva (Qin et al. 2016, Yeri et al. 2017, Godoy et al. 2018). Within the extracellular space, extracellular YRNA (exYRNA) levels are dysregulated in the blood of patients with heart disease, cancer and endotoxemia (Repetto et al. 2015, Guglas et al. ^2020^). Furthermore, cells exposed to immune‐activating stimuli such as LPS can significantly alter their secretion of YRNAs (Hizir et al. 2017, Driedonks et al. 2018). But exYRNAs appear to be more than just passive biomarkers of disease, as they have been shown to have immunoregulatory properties. In vitro, YRNA‐containing extracellular vesicles (EVs) and immune complexes can induce cytokine secretion, NF‐ΚB pathway modulation and cell death in recipient cells (Hizir et al. 2017, Cambier et al. 2017, Clancy et al. 2010, Li et al. 2022, Chakrabortty et al. 2015). These findings support that YRNAs can instruct intercellular communication during inflammation, but how YRNA distribution within complex biofluids contributes to tissue pathology during disease is incompletely understood.

In this study, we used murine models of airway inflammation to investigate the role of RNY1 in asthma‐like immune responses. We tested (1) how YRNA levels change locally in the lung during tissue inflammation, (2) how exYRNAs are packaged in airway fluid and (3) how YRNAs contribute to the inflammatory signalling in the extracellular space. By defining the packaging and immunological roles of extracellular YRNAs, this study reveals both heterogeneous packaging of YRNA species and YRNA‐dependent intercellular communication by biofluid extracellular particles (EPs).

## 2 Methods

### 2.1 Animals

All animal studies were performed after obtaining approval from the Vanderbilt Institutional Animal Care and Use Committee. RNY1 knockout mice were obtained from GemPharmatech (strain T049875). All other mice were wild‐type C57BL/6J (JAX 000664). Mice were used between 7 and 16 weeks and were housed in a specific pathogen‐free facility. All airway challenges and intraperitoneal injections were performed under isoflurane anaesthesia.

### 2.2 Ovalbumin (OVA) Airway Inflammation Model

A stock solution of 10 mg/mL OVA protein from chicken egg white (Millipore Sigma A5503) was prepared by resuspending 50 mg OVA in 5 mL pharmaceutical‐grade PBS (VWR K812). For sensitization via intraperitoneal injection, 50 µg of OVA per mouse was used. 5 µL of 10 mg/mL OVA was combined with 95 µL pharmaceutical‐grade PBS and 100 µL Imject Alum (ThermoFisher 77161) for 200 µL total volume per mouse. This solution was incubated for 0.5–1 h at room temperature with rocking. Mice were intraperitoneally injected with 200 µL OVA+alum solution on Day ‐9. For the airway challenge, 50 µg of OVA per mouse was used. 5 µL of 10 mg/mL OVA was combined with 15 µL pharmaceutical‐grade PBS for 20 µL total per mouse. Mice were challenged by oropharyngeal aspiration of 20 µL OVA for allergic airway mice or 20 µL PBS vehicle control on Days ‐2, ‐1 and 0. Mice were euthanized 1, 4, 9 or 13 days after the last challenge.

### 2.3 House Dust Mite (HDM) Airway Inflammation Model

A 100 mg/mL stock solution of HDM extract (Greer XPB70D3A25) was prepared by resuspending 159.7 mg extract in 1.597 mL pharmaceutical grade PBS (VWR K812). A 10 mg/mL working stock of HDM extract was prepared by resuspending 40 µL stock solution in 360 µL pharmaceutical‐grade PBS. For oropharyngeal aspiration, 40 µg HDM extract per mouse was used. 4 µL of 10 mg/mL HDM extract was diluted in 36 µL pharmaceutical grade PBS, for 40 µL total volume per mouse. Mice were challenged by oropharyngeal aspiration of 40 µL HDM extract for allergic airway mice or 20 µL PBS vehicle control. Mice were challenged on Days ‐16, ‐14, ‐12, ‐9, ‐7, ‐5, ‐2, ‐1 and 0. Mice were euthanized 1 day after the last challenge.

### 2.4 Airway Cell and exRNA Collection

Euthanized mice were cut in the upper abdomen to expose the diaphragm. An 18‐gauge needle was used to pierce the diaphragm as a secondary method of euthanasia. Mice were then cut peripherally towards the head to expose the trachea. A small incision was made in the trachea to insert a catheter. For RNA collection, the lung was slowly washed with 1 mL of ice‐cold PBS to collect bronchoalveolar lavage fluid (BALF). For BALF cell flow cytometry, the lung was slowly washed with 1 mL of ice‐cold FACS buffer (PBS + 2% FBS + 0.02% sodium azide) to collect BALF. Washing was repeated 2–4 times. BALF washes were then spun at 500 × *g* for 5 min to pellet BALF cells. The BALF cell pellet was used for downstream processing. The supernatant was decanted and spun at 2000 × *g* for 30 min to remove cell debris. The supernatant was decanted again and used for downstream processing.

### 2.5 Lung Tissue RNA Collection

After BALF collection, the lungs were excised from the mouse and placed in FBS‐containing media. Lungs were then washed with PBS. A ∼50 mg piece of lung was cut off, minced and placed in a tube containing 1 mL of Trizol (Invitrogen 15596026). 500 µL of 1 mm soda lime glass beads (Biospec products) were added to each tube. Tubes were beaten in a Mini Beadbeater (Biospec products) for 1 min to homogenize lung tissue. The resulting Trizol/lung tissue mixture was placed in a fresh tube for RNA extraction.

### 2.6 RNA Extraction

All solid samples (cells/tissues) were placed in Trizol Reagent (Invitrogen 15596026). All aqueous samples (Raw BALF, EV_Fx_, Protein_Fx_ SEC fractions) were placed in Trizol LS Reagent (Invitrogen 10296010). RNA was isolated via phenol‐chloroform extraction and isopropanol‐based precipitation. Glycoblue Coprecipitant (Invitrogen AM9516) was added to each sample to improve RNA pellet visibility.

### 2.7 Reverse Transcription

For noncoding RNAs, RNA was reverse transcribed using the Mir‐X miRNA First Strand Synthesis Kit (Takara 638315) according to the manufacturer's instructions. For mRNAs, RNA was reverse transcribed using Superscript IV First Strand Synthesis System (Invitrogen 18091050) according to the manufacturer's instructions.

### 2.8 qPCR

qPCR reactions consisted of cDNA, forward/reverse primers (see Table S1), and FastStart Universal SYBR Green Master ROX (Roche 04913914001). qPCR reactions were run under the following cycling conditions: 95°C for 10 min followed by 40 cycles of 95°C for 15 s and 61°C for 30 s.

For intracellular mRNA, Ct values were normalized to the average Ct of three housekeeping genes (Arpc2, Cltc, Rab14). Housekeeping genes were selected from the bulk RNA sequencing data under the following conditions: PBS versus RNY1^+/+^ Protein_Fx_ |Log2FC| < 0.2, RNY1^+/+^ Protein_Fx_ versus RNY1^−/−^ Protein_Fx_ |Log2FC| <0.2, CPM >100.

### 2.9 Flow Cytometry

BALF cells were incubated in ACK lysis buffer to remove red blood cells and counted using a haemocytometer. Cells were then incubated with Fc block (Invitrogen 14‐0161‐85) at a 1:1000 dilution and Fixable Viability Dye eFluor 780 (Invitrogen 65‐0865‐14) at a 1:500 dilution. After washing once with FACS buffer, BALF cells were incubated with the following antibodies at a 1:8 dilution: PE‐CD11b (Tonbo 50‐0112‐U100), PerCP Cy5.5‐Siglec F (BD Biosciences 565526), PE Cy7‐F4/80 (Tonbo 60‐4801‐U100), FITC‐Ly6G (Biolegend 127606), VioletFluor450‐CD45 (Tonbo 75‐0451‐U100) and APC‐CD11c (Invitrogen 17‐0114‐82). Lymph node cells were used for unstained and single‐stained compensation controls. Flow cytometry was performed using the FACSCanto II flow cytometer (BD Biosciences). Data was analysed using FlowJo software. Immune cell subset abundance was calculated by determining the proportion of gated immune cell populations relative to total live cells acquired by flow cytometry. This proportion was multiplied by the total BALF cell count to calculate cells per airway.

### 2.10 RNase Sensitivity Assays

Unconcentrated BALF (250 µL/sample), EV_Fx_ (5 × 10^9^–1 × 10^10^ particles/sample) and Protein_Fx_ (200 µg/sample) were incubated with 40 µg/mL RNase A (Thermo Scientific EN0531) for 15 min at 37°C. Proteinase K treated samples were incubated with 500 µg/mL Proteinase K (Fisher Scientific BP‐1700‐100) for 1 h at 37°C prior to RNase treatment. Triton X‐100 treated samples were vortexed for 30 s in 0.1% Triton X‐100 (Sigma T8787) solution prior to RNase treatment. Samples subjected to all three treatments were incubated with Triton X‐100, then Proteinase K, then RNase. Samples treated with Proteinase K received two rounds of RNase treatment to ensure adequate RNase degradation occurred in the presence of Proteinase K.

### 2.11 Size Exclusion Chromatography (SEC)

BALF was concentrated using an Amicon ultracentrifugal filter with a 100 kDa pore size (Millipore UFC 910024). The retentate was brought to a final volume of 500 µL with PBS and fractionated using a 35 nm qEV Original v2 size exclusion column (Izon) according to the manufacturer's instructions. Columns were equilibrated and buffered with 0.2 µm‐filtered PBS. After a 2.5 mL void volume, fractions were collected in 400 µL increments.

### 2.12 EV_Fx_ and Protein_Fx_ Preparation

BALF was collected, processed and SEC‐fractionated as described above. SEC fractions were pooled for EV‐enrichment (fractions 1–4) and Protein‐enrichment (fractions 9–20). Pooled fractions were concentrated using an Amicon ultracentrifugal filter with a 10 kDa pore size (Millipore UFC 901024) and used for downstream analyses.

### 2.13 Nanoparticle Tracking Analysis

The concentration and size of EVs were analysed by nanoparticle tracking analysis (NTA) using a ZetaView (ParticleMetrix) with the following parameters: Laser wavelength = 488 nm, filter wavelength = scatter, sensitivity = 70, Shutter = 100. EVs were diluted 1000–10,000 times in PBS, and measurements were performed using 1 cycle and 11 camera positions. Data were analysed using ParticleMetrix's NTA software.

### 2.14 Western Blot

Western blots were performed with the Jess automated Western blot system (BioTechne) according to the manufacturer's instructions for Western blot + total protein quantification replex assay. 600 ng of protein lysate was used per sample. Samples were stained with the following antibodies at a 1:20 dilution: anti‐TSG101 (Abcam ab30871), anti‐CD9 (Abcam ab307085) and anti‐Calnexin (Abcam ab22595). Data were analysed using Compass for SW Western blot analysis software (BioTechne).

### 2.15 Bone Marrow‐derived Macrophage Culture

All mice used for bone marrow‐derived macrophage (BMDM) culture were derived from female mice. Bone marrow cells were isolated from C57BL/6J murine femurs and tibias. Bone marrow cells were cultured for 5 days in RPMI media containing 10% FBS and 20% L929 fibroblast‐conditioned media. On Day 6, BMDMs were seeded in 96‐well plates at 200,000 cells/well and switched to RPMI media containing 10% FBS. BMDMs were used for experiments on Day 7 of culture.

### 2.16 Macrophage Treatments With Airway EV_Fx_ and Protein_Fx_

BALF EV_Fx_ and Protein_Fx_ were collected and stored at 4°C for 5 days. Before treatment, BMDMs were washed 3× with PBS and resuspended in Opti‐MEM media without FBS. EV_Fx_ from RNY1^+/+^ and RNY1^−/−^ BALF were added to BMDM cultures at equal particle concentrations (3.5 × 10^7^ particles/µL). Protein_Fx_ from RNY1^+/+^ and RNY1^−/−^ BALF were added to BMDM cultures at equal protein concentrations (0.75 µg/µL).

### 2.17 RNA Sequencing Sample Preparation

BMDMs were lysed in 70 µL DNA/RNA shield. Lysates were vortexed briefly to reduce sample viscosity. RNA extraction and sequencing was performed by Plasmidsaurus according to their protocol for Illumina RNA sequencing. Briefly, polyadenylated mRNA transcripts were enriched and reverse transcribed into cDNA using oligo(dT) primers containing sample barcodes, unique molecular identifiers (UMIs) and Illumina Read 1 adapter sequences. Double‐stranded cDNA was subsequently generated by second‐strand synthesis. cDNA libraries were fragmented and tagged by tagmentation, which simultaneously incorporated Illumina Read 2 adapter sequences. Final sequencing libraries were amplified by PCR to incorporate unique dual indices (i5 and i7) and Illumina P5/P7 flow cell adapter sequences.

### 2.18 RNA Sequencing Bioinformatic Analysis

Data processing and analysis was performed by Plasmidsaurus. Quality of the fastq files was assessed using FastQC v0.12.1. Reads were then quality filtered using fastp v0.24.0 with poly‐X tail trimming, 3' quality‐based tail trimming, a minimum Phred quality score of 15, and a minimum length requirement of 50 bp. Quality‐filtered reads were aligned to the reference genome using STAR aligner v2.7.11 with non‐canonical splice junction removal and output of unmapped reads, followed by coordinate sorting using samtools v1.22.1. PCR and optical duplicates were removed using UMI‐based deduplication with UMIcollapse v1.1.0. Alignment quality metrics, strand specificity and read distribution across genomic features were assessed using RSeQC v5.0.4 and Qualimap v2.3, with results aggregated into a comprehensive quality control report using MultiQC v1.32. Gene‐level expression quantification was performed using featureCounts (subread package v2.1.1) with strand‐specific counting, multi‐mapping read fractional assignment, exons and 3′ UTR as the feature identifiers, and grouped by gene_id. Final gene counts were annotated with gene biotype and other metadata extracted from the reference GTF file. Sample‐sample correlations for the sample‐sample heatmap and PCA were calculated on normalized counts (TMM, trimmed mean of M‐values) using Pearson correlation. Differential expression was done with edgeR v4.0.16 using standard practice, including filtering for low‐expressed genes with edgeR::filterByExpr with default values. Functional enrichment, when available, was performed using gene set enrichment analysis with gseapy v0.12 using the MSigDB Hallmark gene set. All data availble on the Gene Expression Omnibus (GSE333855).

## 3 Results

### 3.1 Extracellular RNY1 Levels Increase During Airway Inflammation

**FIGURE 1:**
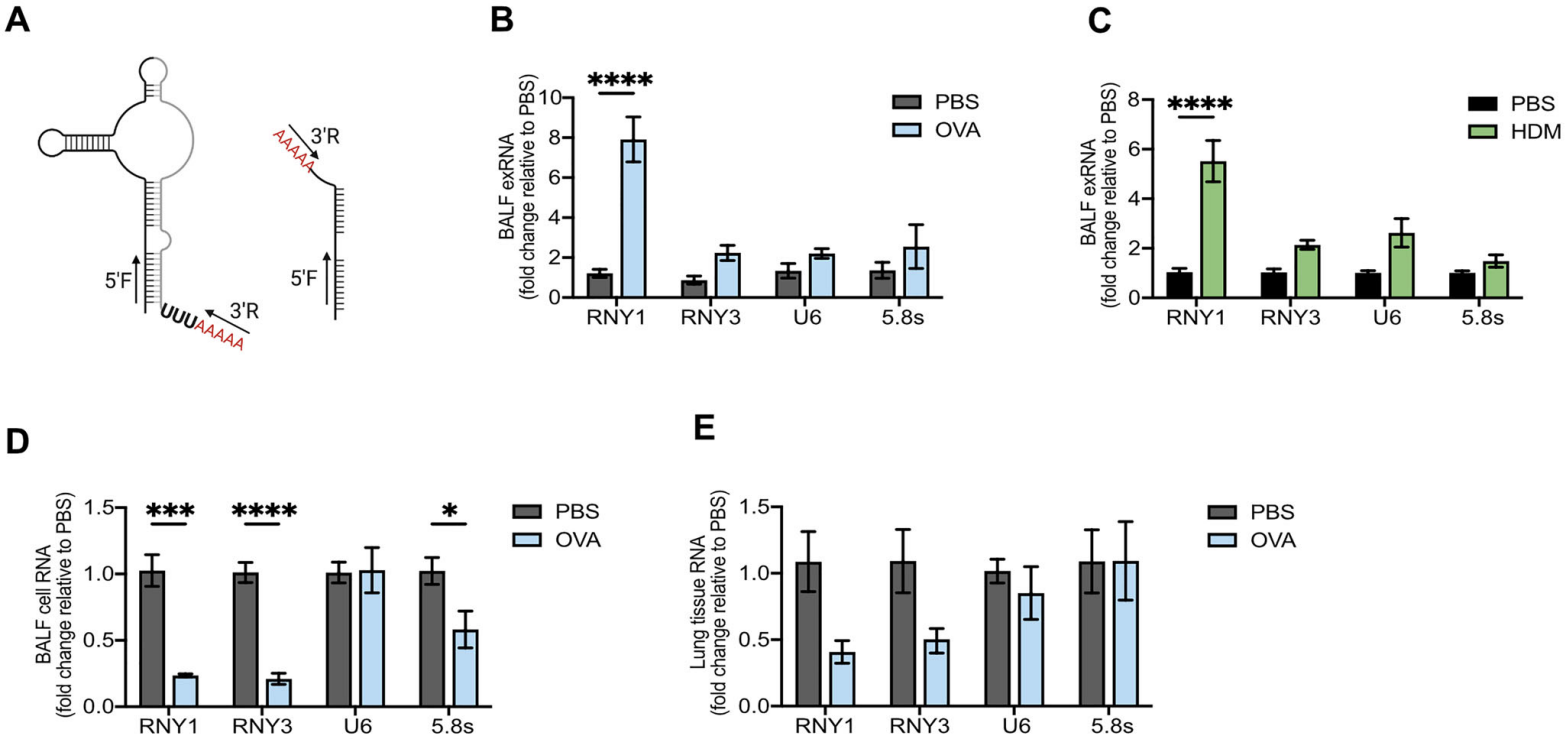
Extracellular YRNA levels increase during airway inflammation. (A) YRNA qPCR schema. After BALF RNA extraction, RNAs were polyadenylated and reverse transcribed. Each primer set has a primer that detects the 5' end of the YRNA (5'F) and a universal reverse primer (3'R) that binds to the added polyadenylated tail. The primer sets detect both full‐length and 5′ fragments of YRNAs. (B, C) RNA qPCR of murine BALF exRNA 1 day after the last airway challenge in the OVA (B) and HDM (C) models of airway inflammation. Data is expressed as the fold change (2^−ΔCt^, normalized to PBS‐challenged control average Ct). Two‐way ANOVA with Bonferroni. *N* = 4–10 from 2+ independent experiments. (D and E) RNA qPCR of murine BALF cells (D) and lung tissue (E) 1 day after inducing the OVA model of airway inflammation. Data are expressed as the fold change (2^−ΔΔCt^, normalized to beta actin Ct and PBS‐challenged control average Ct). Two‐way ANOVA with Bonferroni. *N* = 5 from two independent experiments. For all panels: *p ≤ 0.05, **p ≤ 0.01, ***p ≤ 0.001, ****p ≤ 0.0001. Bars represent the mean ± standard error of the mean. BALF, bronchoalveolar lavage fluid.

Circulating exYRNA levels increase in autoimmune disorders, cancer and cardiovascular disease (Zhao et al. 2024, Mori et al. 2019, Quinn et al. 2015, Godoy et al. 2018, Baglio et al. 2016). Yet, it is not well understood how extracellular YRNA dynamics change at local tissue sites during inflammation. The lung offers an excellent system to investigate tissue immune responses with an accessible biofluid, which we and others have shown contains exRNAs that fluctuate in response to inflammation (Pua et al. 2019, Levänen et al. 2013, Song et al. 2022). To investigate how exYRNAs levels change during airway inflammation, we used a model of asthma where mice are first sensitized to the ovalbumin (OVA) antigen, then challenged in the airway to induce a local allergic response (Figure S1A). To quantify levels of YRNAs, we performed qPCR for the two mouse YRNAs (RNY1 and RNY3) in total bronchoalveolar lavage fluid (BALF) cleared of cells and debris by serial centrifugation (500*g* × 5 min, 2000*g* × 30 min). Primers were designed to capture both full‐length and fragmented isoforms, as YRNAs exist in both forms in the extracellular space (Figure 1A) (Driedonks et al. 2020, Driedonks et al. 2018, Clancy et al. 2010, Driedonks and Nolte‐T'Hoen 2019, Reed et al. 2013). We observed an ∼8‐fold increase in RNY1 levels in OVA‐challenged mice when compared to mice challenged with PBS vehicle control. RNY3 and several other noncoding RNAs were not significantly increased in the extracellular space (Figure 1B). This preferential increase in BALF exRNY1 levels also occurred in house dust mite (HDM)‐challenged mice, a second model of sterile allergen‐induced lung inflammation (Figure 1C, Figure S1B). To determine if increased RNY1 levels could result from increased cellular RNY1 levels, we quantified this YRNA in BALF cells and lung tissue. Interestingly, there was a significant decrease in total RNY1 expression in BALF cells and a trend towards decreased RNY1 levels in lung tissue with airway inflammation (Figure 1D,E). These results show that RNY1 levels in the extracellular space selectively increase during airway inflammation with a concomitant decrease in intracellular YRNA expression.

### 3.2 Extracellular RNY1 Kinetics Correlate With Immune Cell Infiltration During Airway Inflammation

**FIGURE 2:**
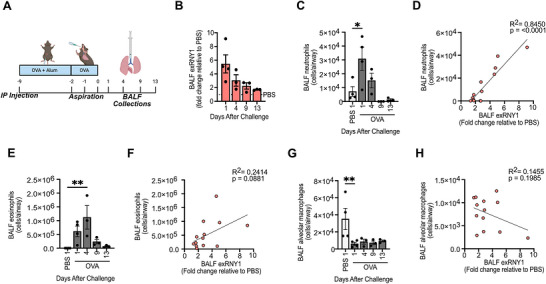
exRNY1 levels correlate with neutrophilic inflammation in the lung. (A) BALF collection timepoints after OVA airway challenge. BALF was collected 1, 4, 9 and 13 days after the last challenge. (B) YRNA levels in murine BALF collected 1, 4, 9 and 13 days after OVA‐induced airway inflammation. Data are expressed as the fold change (2^−ΔCt^, normalized to PBS‐challenged control average Ct). *N* = 3–4 from one independent experiment. (C, E, G) BALF neutrophil (C), eosinophil (E) and alveolar macrophage (G) counts from murine BALF collected 1, 4, 9 and 13 days after OVA‐induced airway inflammation were determined via flow cytometry. One‐way ANOVA with Bonferroni. *N* = 3–4 from one independent experiment. (D, F, H) Linear regression analyses of BALF YRNA fold changes versus eosinophil (D), neutrophil (F) and alveolar macrophage (H) numbers over time. For all panels: *p ≤ 0.05, **p ≤ 0.01, ***p ≤ 0.001, ****p ≤ 0.0001. Bars represent the mean ± standard error of the mean. *N* = 3–4 mice from each timepoint. BALF, bronchoalveolar lavage fluid; OVA, ovalbumin.

Since we observed an increase in exRNY1 levels during airway inflammation, we wanted to determine how exRNY1 fluctuates with transient and resident immune cell populations in the lung over time. Therefore, we measured exRNY1 and immune cell levels 1, 4, 9 and 13 days after the induction of airway inflammation (Figure 2A). RNY1 levels peaked 1 day after the last airway allergen challenge, then decreased over time (Figure 2B). To test whether the levels of RNY1 correlated with specific immune cell populations over the course of inflammation, we measured eosinophil, neutrophil and macrophage cell dynamics in BALF using flow cytometry (Figure S2). We observed a strongly significant correlation of extracellular RNY1 levels with neutrophils (*R* ^2 ^= 0.85), which peaked and then rapidly declined after the last airway challenge (Figure 2C,D). We did not see a significant correlation of RNY1 levels with eosinophils, which peaked 4 days after the last airway challenge (Figure 2E,F). There was no correlation between extracellular RNY1 levels and macrophages, which are a resident immune cell population whose numbers contracted slightly in this inflammatory model (Figure 2G,H). Together, these data show that during inflammation, increases in exRNY1 levels positively correlate with rapidly responding neutrophil infiltration within the local tissue microenvironment.

### 3.3 Extracellular YRNAs Are Protected From Degradation by Lipids and Proteins in Airway Fluid

**FIGURE 3:**
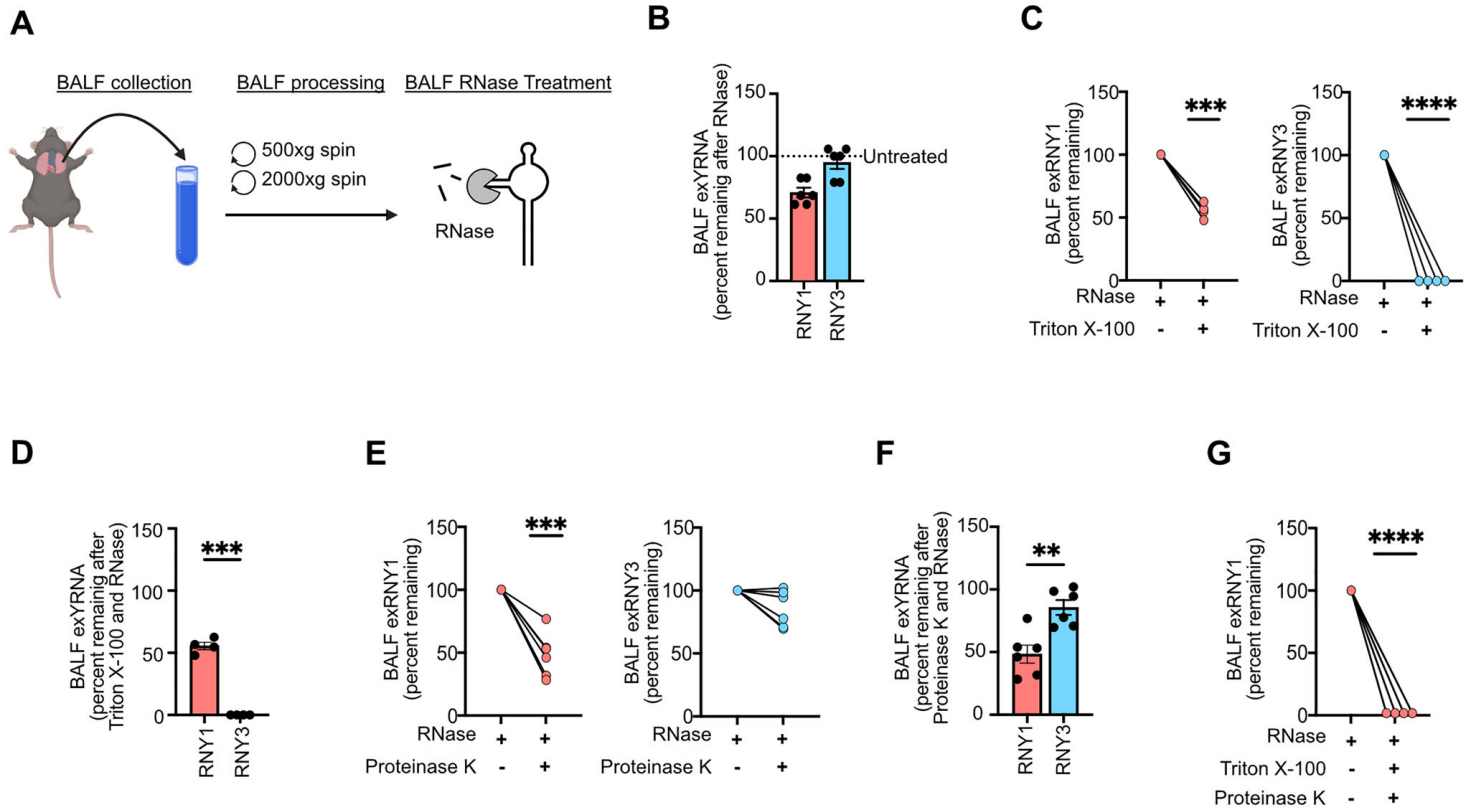
RNY1 is protected from degradation by lipid and protein‐containing structures during airway inflammation. (A) RNase sensitivity experimental workflow. (B) YRNA qPCR of RNase‐treated BALF. Data are expressed as the percentage of remaining YRNA compared to an untreated control. Welch's *t*‐test. *N* = 3–6 mice from 2 to 3 independent experiments. One RNY3 value was removed based on the Grubb's outlier test. (C) qPCR of RNY1 (left) and RNY3 (right) from Triton X‐100 + RNase‐treated BALF. Data are expressed as the percentage of remaining YRNA compared to an RNase‐only treated control. One sample student's *t*‐test. *N* = 4 mice from two independent experiments. (D) Direct comparison of RNY1 and RNY3 percent remaining after Triton X‐100 + RNase treatment (from B). Welch's *t*‐test. (E) qPCR of RNY1 (left) and RNY3 (right) Proteinase K + RNase‐treated BALF. Data are expressed as the percentage of remaining YRNA compared to an RNase‐only treated control. One sample student's *t*‐test. *N* = 6 mice from two independent experiments. (F) Direct comparison of RNY1 and RNY3 percent remaining after Proteinase K + RNase treatment (from E). Welch's *t*‐test. (G) RNY1 qPCR of Triton X‐100 + Proteinase K + RNase‐treated BALF. Data are expressed as the percentage of remaining RNY1 compared to an RNase‐only treated control. One sample student's *t*‐test. *N* = 5 mice from two independent experiments. For all panels: *p ≤ 0.05, **p ≤ 0.01, ***p ≤ 0.001, ****p ≤ 0.0001. Bars represent the mean ± standard error of the mean. BALF, bronchoalveolar lavage fluid; OVA, ovalbumin.

Understanding how RNAs are packaged in the extracellular space is necessary to determine their sources, secretion pathways, stability and functions in cellular communication. To test whether exYRNAs are stably packaged in the inflamed airway, we performed an RNAse sensitivity assay (Figure 3A). Pre‐extracted RNA from BALF was RNAse‐sensitive, suggesting that that naked YRNA sequences are not protected by post‐transcriptional modifications or secondary structure (Figure S3A). In contrast, ∼75% of exRNY1 and ∼95% of exRNY3 were RNAse‐resistant in native BALF during airway inflammation (Figure 3B). There was no statistically significant difference in YRNA RNAse sensitivity between PBS‐ and OVA‐challenged mice (Figure S3B). Together, these results demonstrate that exYRNAs are protected from degradation by extracellular structures.

Extracellular RNAs are often protected inside lipid‐bilayer‐enclosed structures such as EVs in biofluids. To test if YRNAs are packaged inside lipid‐protected structures, we treated BALF with the non‐denaturing detergent Triton X‐100 and evaluated YRNA levels after RNase treatment. In the inflamed airway, both RNY1 and RNY3 were significantly degraded after detergent treatment, consistent with their being present in lipid‐containing structures (Figure 3C). Importantly, detergent did not disrupt the ability of RNAse to degrade pre‐extracted BALF YRNAs, demonstrating that the observed protection was not the result of enzyme activity interference (Figure S3C). Interestingly, we observed that significantly more RNY1 remained RNAse‐resistant after Triton X‐100 treatment than RNY3 (Figure 3D). Therefore, we evaluated whether a subset of RNY1 in the extracellular space may instead be protected from degradation by proteins. When BALF was treated with Proteinase K + RNase, RNY1 but not RNY3 partially degraded (Figure 3E,F). Proteinase K did not disrupt the ability of RNAse to degrade YRNAs from extracted BALF RNA (Figure S3D). Co‐treatment of BALF with detergent, Proteinase K and RNAse resulted in the complete degradation of RNY1, suggesting heterogeneous compartmentalization of RNY1 in the BALF (Figure 3G). Finally, we observed no statistically significant differences in YRNA RNAse sensitivity patterns in BALF treated with Triton X‐100 and Proteinase K from PBS‐ or OVA‐ challenged mice (Figure S3E,F), demonstrating that YRNAs maintain similar protection patterns with and without airway inflammation.

### 3.4 RNY1 Is Present in EV‐Enriched and Protein‐Enriched Fractions During Airway Inflammation

**FIGURE 4:**
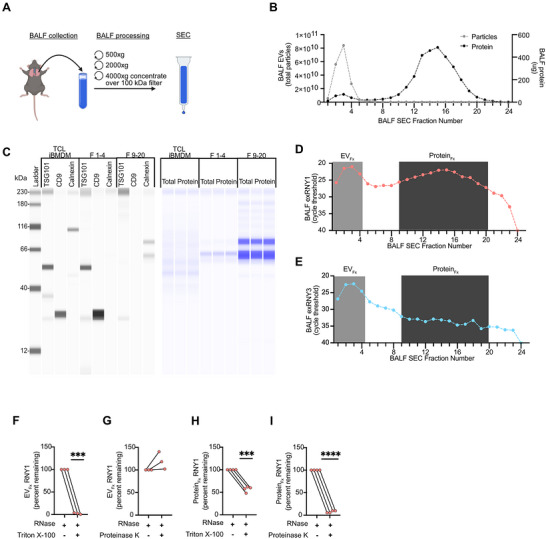
RNY1 is stably packaged in EV‐ and protein‐enriched fractions during airway inflammation. (A) BALF SEC experimental workflow. (B) Particle and protein quantification in BALF SEC fractions. *N* = 1 from one independent experiment. (C) Western blot of pooled BALF SEC fractions 1–4 and 9–20. Western blot was performed using a capillary‐based electrophoresis system with virtual, blot‐like representations of chemiluminescent signals for each antibody, or a biotin label for total protein. Immortalized BMDMs were used as a technical control for calnexin detection. *N* = 1 from one independent experiment. (D, E) RNY1 (D) and RNY3 (E) qPCR cycle threshold values in BALF SEC fractions. (F, G) RNY1 qPCR of EV_Fx_ treated with Triton X‐100 + RNase (F) or Proteinase K + RNase (G). Data are expressed as the percentage of remaining YRNA compared to an RNase‐only treated control. One sample student's *t*‐test. *N* = 5 from two independent experiments. (H, I) RNY1 qPCR of Protein_Fx_ treated with Triton X‐100 + RNase (H) or Proteinase K + RNase (I). Data are expressed as the percentage of remaining RNY1 compared to an RNase‐only treated control. One sample student's *t*‐test. *N* = 5 from two independent experiments. For all panels: *p ≤ 0.05, **p ≤ 0.01, ***p ≤ 0.001, ****p ≤ 0.0001. Bars represent the mean ± standard error of the mean. Open circles indicate no detection. BALF, bronchoalveolar lavage fluid; BMDM, bone marrow‐derived macrophages; EV_Fx_, EV‐enriched fraction; Protein_Fx_, protein‐enriched fraction.

To further characterize how YRNAs are protected in airway fluid during inflammation, we fractionated BALF using size exclusion chromatography (SEC) (Figure 4A). As anticipated, particles with a size of ∼110 nm were enriched in fractions 1–4 (EV_Fx_), while total protein was enriched in fractions 9–20 (Protein_Fx_) (Figures 4B, S4A). In addition, the EV_Fx_ contained the luminal EV marker Tsg101 and the transmembrane EV marker CD9 while the Protein_Fx_ did not (Figure 4C). Neither fraction contained Calnexin, a proxy marker for cellular contaminants (Figure 4C). Together, these data demonstrate that BALF can be partitioned into EV‐ and protein‐rich fractions by SEC.

Next, we investigated the distribution of YRNAs in the EV_Fx_ and the Protein_Fx_. By performing YRNA qPCR across SEC fractions, we observed two peaks of RNY1 detection, one centered in the EV_Fx_ and one centred in the Protein_Fx_ (Figure 4D). In contrast, RNY3 levels only peaked in the EV_Fx_ (Figure 4E). To test how YRNAs are protected in each of these fractions, we performed RNAse sensitivity assays. Within the EV_Fx_, RNY1 and RNY3 were only susceptible to RNAse degradation after Triton X‐100 treatment (Figures 4F,G, S4B,C). Within the Protein_Fx_, RNY1 was susceptible to RNAse degradation after proteinase treatment and partially susceptible after Triton X‐100 treatment (Figure 4H,I). This observation suggests that RNY1 in the Protein_Fx_ may be present in heterogeneous structures, including non‐vesicular extracellular particles (NVEPs), which contain both protein and lipids (i.e. lipoprotein particles, supermeres or exomeres) in the airway fluid (Welsh et al. 2024, Xu et al. 2025). In total, this data shows that BALF RNY1 is stably associated with heterogeneous EPs across distinct extracellular compartments.

### 3.5 RNY1 Modulates Macrophage Transcriptional Programming by Airway Fluid During Inflammation

**FIGURE 5:**
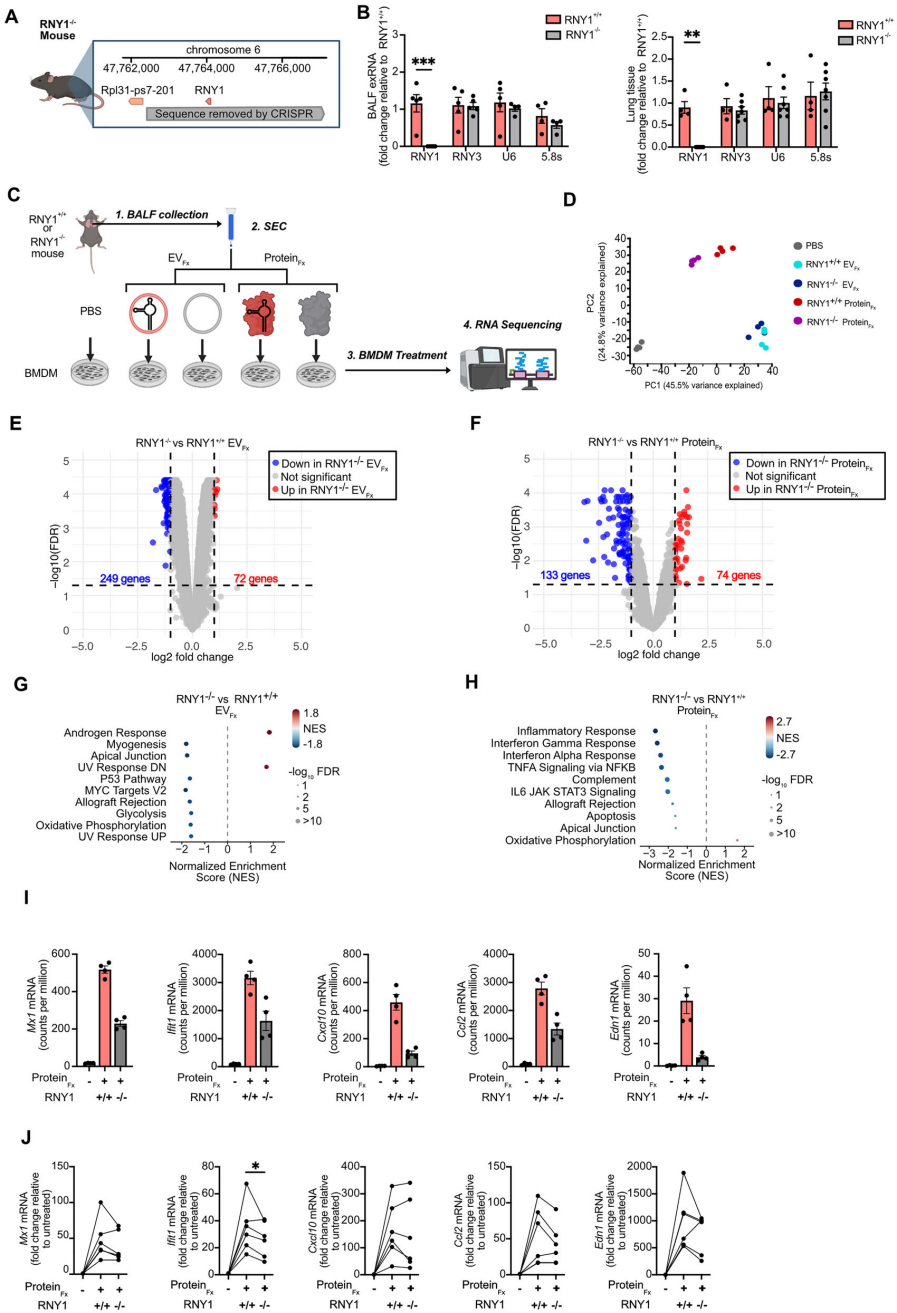
RNY1 fine‐tunes macrophage transcriptional programming by BALF Protein_Fx_. (A) Chromosomal region of CRISPR deletion in *RNY1^−/−^* mice. (B) RNA qPCR of BALF (left) and lung tissue (right) RNAs derived from *RNY1^+/+^* and *RNY1^−/−^* mice. Data are expressed as the mean fold change compared to the average *RNY1^+/+^* control value. *N* = 5 from two independent experiments. Welch's *t*‐test. (C) RNA sequencing experiment workflow. (D) Principal component analysis of BMDM transcriptional composition by treatment group. Colors represent treatment groups (*n* = 4/group). (E, F) Volcano plots of DEGs in RNY1^−/−^ EV_Fx_‐ treated BMDMs (E) and Protein_Fx_‐treated BMDMs (F) compared to RNY1^+/+^. Genes with CPM <3 were removed from analysis. Significance cutoffs are FDR > 0.05 and log_2_ fold change > 1. (G) Top 10 pathways identified in gene set enrichment analysis (GSEA) using the Hallmark gene set. Dots represent the ‐log_10_ FDR and NES of pathways in RNY1^−/−^ Protein_Fx_‐treated BMDMs compared to RNY1^+/+^ Protein_Fx_‐treated BMDMs. (H) Top 10 pathways identified in gene set enrichment analysis using the Hallmark gene set. Dots represent the ‐log_10_ FDR and NES of pathways in RNY1^−/−^ EV_Fx_‐treated BMDMs compared to RNY1^+/+^ EV_Fx_‐treated BMDMs. (I) Candidate interferon‐associated DEG read counts in Protein_Fx_‐treated BMDMs. (I) Candidate interferon‐associated DEG qPCR. Cycle threshold values were normalized to housekeeping gene control averages (Figure S6H,I). Data are expressed as the fold change (2^−ΔΔCt^) relative to the PBS‐treated BMDM control. *N* = 6 from two independent experiments. Ratio paired *t*‐test performed on −ΔCt values. For all panels: *p ≤ 0.05, **p ≤ 0.01, ***p ≤ 0.001, ****p ≤ 0.0001. Bars represent the mean ± standard error of the mean. BALF, bronchoalveolar lavage fluid; BMDM, bone marrow‐derived macrophage; EV_Fx_, EV‐enriched fraction; Protein_Fx_, protein‐enriched fraction.

Given exRNY1's heterogeneous distribution in BALF, we next wanted to explore how distinct RNY1‐containing BALF compartments guide immune responses by utilizing a CRISPR‐mediated RNY1 knockout mice (Figure 5A). Mice with both alleles of the RNY1 locus knocked out (*RNY1^−/−^*) were born at normal Mendelian frequencies and had no grossly observed phenotypic defects (data not shown). These mice lacked expression of RNY1 in BALF and lung tissue, while RNY3 and other noncoding RNA levels remained constant (Figure 5B). During OVA‐induced airway inflammation, *RNY1^−/−^* mice had normal recruitment of eosinophils, neutrophils, CD4^+^ T cells and B cells to the lung, indicating that the ability to mount an inflammatory response to allergen challenge remained intact despite systemic loss of RNY1 expression (Figure S5A‐D). We also observed no differences in particle and protein fractionation during SEC (Figure S5E). Like *RNY1^+/+^* mice, *RNY1^−/−^* BALF EV_Fx_ contained the EV markers TSG101 and CD9, and these markers were absent in the Protein_Fx_ (Figure S5F). BALF EV_Fx_ particle number, particle size and protein amount remained constant between *RNY1^−/−^* and *RNY1^+/+^* mice (Figure S5G‐J). Additionally, there was no significant change in the total protein content of the Protein_Fx_ (Figure S5J).

Macrophages are an ideal model system to investigate complex cellular responses to EPs, as they are phenotypically plastic cells that adopt diverse transcriptional profiles in response to extracellular stimuli from the local microenvironment. To test how RNY1 contributes to the function of EPs in airway fluid during inflammation, we treated bone marrow‐derived macrophages (BMDMs) with EV_Fx_ and Protein_Fx_ isolated from OVA‐challenged mice and performed bulk RNA sequencing (Figure 5C, Table S2). EV_Fx_ was dosed at a total of 3.5 × 10^10^ particles/mL. Protein_Fx_ was dosed at 0.75 mg/mL total protein. Each of these is less than our estimate of the physiologic concentration of EVs and protein in airway lining fluid (Figure S6A,B). Principal component analysis of the data set showed tight clustering by treatment condition, as well as a large amount of variance between untreated BMDM and each treatment condition (Figure 5D). Both the EV_Fx_ and Protein_Fx_ induced large transcriptional responses in macrophages, with >2000 differentially expressed genes for each treatment condition (Figure S6C,D, Tables S3 and S4). Additionally, GSEA analysis revealed that the EV_Fx_‐ and Protein_Fx_‐treated macrophages were enriched in classical pro‐inflammatory immune response pathways, indicating that both BALF compartments are capable of immune signalling (Figure S6E,F, Tables S5 and S6).

To investigate the contribution of RNY1 to programming of macrophages by BALF, we next compared treatment with *RNY1^+/+^* and *RNY1^−/−^* fractionated airway fluid. Although we did observe differences between EV_Fx_‐treated groups, the magnitude of these changes were modest, and top differential pathways were not related to inflammatory immune responses (Figure 5E,G, Tables S7 and S8). In contrast, we observed larger effect sizes in differentially expressed genes (DEGs) between macrophages treated with *RNY1^+/+^* versus *RNY1^−/−^* Protein_Fx_. Gene set enrichment analysis of DEGs highlighted several immune‐ and inflammation‐related pathways as de‐enriched in the *RNY1^−/−^* treated BMDMs, with two of the top three pathways related to interferon responses (Figure 5F,H, Table S9 and S10). To validate our RNA sequencing findings, we selected candidate genes associated with the interferon response that were differentially expressed and performed qPCR (Figure 5I). Although our results showed some variability between biologic replicates, there was a consistent trend toward reduced induction of interferon‐associated transcripts in *RNY1^−/−^* Protein_Fx_‐treated BMDMs (Figures 5J and S6G‐I). These results demonstrate that RNY1 can contribute to airway fluid‐mediated programming of macrophages.

## 4 Discussion

The levels and packaging of exRNAs are dynamic in extracellular fluids. Prior studies have shown that circulating YRNA levels in blood change during disease (Repetto et al. 2015, Dhahbi et al. 2014). Our work expands these studies by investigating localized biofluid exYRNA levels in conjunction with associated cell and tissue dynamics during sterile lung inflammation. We observed a large, selective increase in exRNY1 levels while cell and tissue RNY1 levels decreased. This observation opens new opportunities to explore the interconnectivity of intracellular and extracellular YRNAs in vivo to test how changes in cellular composition, RNA secretion, transcription rates and RNA degradation during inflammation impact exRNA secretion. Key next steps include determining the cellular origin of RNY1 in airway fluid and cell‐specific YRNA secretion patterns in response to inflammatory stimuli.

Additionally, this work assays YRNA levels over time and demonstrates that exRNY1 levels strongly correlate with neutrophil levels in the airway. Neutrophilic inflammation is common in asthma subsets that are often severe and refractory to current treatments, making identifying novel biomarkers and drivers of neutrophilic lung inflammation in this disease particularly important Ray et al. 2017). Cell‐type specific RNY1 deletions or in vivo RNA tracing could test whether neutrophils are the source of RNY1 in the lung or instead may be called as part of a response program that includes exYRNA production from other cell types. Understanding the causality of the RNY1‐neutrophil correlation will deepen our understanding of YRNA dynamics as they relate to disease processes.

exRNAs can exist outside the cell as free RNAs, as EV cargos, in NVEPs, and as part of protein complexes. Prior work has identified EP‐associated exYRNA in cell‐conditioned media and biofluids (Wei et al. 2017, Driedonks et al. 2020, Driedonks et al. 2018, Cambier et al. 2017, Driedonks and Nolte‐T'Hoen 2019, Robbins et al. 2019, Driedonks et al. 2024). By combining SEC with EP characterization methods, we observed distinct extracellular compartmentalization of individual YRNAs. Specifically, RNY1 was distributed across the two classical SEC elution profiles, the EV‐enriched and the protein‐enriched fractions, whereas RNY3 was detected exclusively in EV‐enriched fractions. These findings expand our current understanding of multimodal exRNA distribution in complex biofluids. Additionally, this work motivates future investigation into how YRNAs are distributed across more defined EP subpopulations. By using orthogonal separation approaches and techniques such as RNA‐baited mass spectrometry, we can further classify the subtype of EV, NVEP and/or protein that carries YRNA and integrate that knowledge into our understanding of exYRNA dynamics during disease.

Previous studies have shown that RNY1‐containing EPs can instruct cellular immune responses. The strongest published record of this phenomenon is that EPs and their YRNA cargoes can provide instructing signals to activate macrophages (Tang et al. 2022, Kang et al. 2024). Macrophage activation by both pathogen‐derived and endogenous signals induces rapid transcriptional remodelling of these cells and is critical for host defence, tissue damage during inflammation and reparative functions (Yan et al. 2024, Luo et al. 2024, Roszer et al. 2015). By using macrophages as a model system, we revealed that distinct RNY1‐containing BALF compartments can guide immune responses. EV‐enriched and protein‐enriched airway fluid fractions induced numerous changes to the macrophage transcriptome associated with pro‐inflammatory immune responses. Identifying immunological activity in these compartments provides a framework to resolve EP‐specific immunological communication mechanisms within a single biofluid. Through further characterization of BALF compartments and intercompartment‐normalized dosing, we can further understand how EP‐subtypes differentially induce immune activation in recipient cells.

To build on our previous observation, we wanted to further investigate RNY1's role in BALF EP‐mediated transcriptional reprogramming. This type of investigation has been historically difficult to assess in vivo, as YRNAs are ubiquitously expressed in the intracellular and extracellular space (Driedonks et al. 2024). Here, we generated and characterized an RNY1‐deficient mouse model and used it to investigate how loss of RNY1 alters extracellular compartment‐mediated immune signalling. We observed that the protein‐enriched fraction from *RNY1^−/−^* mice induced less robust pro‐inflammatory and Interferon pathway responses. These findings align with prior reports that YRNA‐associated EPs instruct immunological functions in recipient cells such as cytokine secretion, apoptosis and autoimmune inflammation (Hizir et al. 2017, Cambier et al. 2017, Li et al. 2022). Additional work is needed to determine whether the YRNA itself is initiating signalling from this biofluid, or whether it acts indirectly to modulate other signalling molecules in the extracellular space. Key next steps to fully understand this signalling axis involve investigating the global consequences of genetic RNY1 removal, such as unbiased probing of the intracellular and extracellular proteome, lipidome and transcriptome.

An important consideration for interpreting this study is the structural heterogeneity of extracellular RNY1 itself. RNY1 exists both as a full‐length transcript and as multiple processed fragments, each of which may possess distinct extracellular localization patterns or biological activities. Because our qPCR detection strategy requires an intact 5′ end but is otherwise agnostic to 3′ end length, the measurements presented here likely capture a mixture of RNY1 isoforms. Similarly, the RNY1‐deficient mouse model globally eliminates total RNY1 expression and therefore cannot distinguish between roles for full‐length and fragmented species. Future studies incorporating isoform‐resolved sequencing and structure‐sensitive detection approaches will be important for determining how specific RNY1 species contribute to compartmentalization and immune signalling.

YRNAs are emerging as important mediators of intercellular communication during inflammation and immune responses. This work identifies RNY1 within both EV‐enriched and protein‐enriched extracellular compartments in airway lining fluid, demonstrates that these compartments possess immunomodulatory activity, and establishes a genetic framework for interrogating RNY1‐dependent extracellular signalling in vivo. Together, these findings expand our understanding of YRNA biology and provide a foundation for future studies investigating how YRNAs shape intercellular communication.

## Author Contributions

**Cherie E. Saffold**: conceptualization (lead), methodology (lead), data acquisition (lead), data analysis (lead), data visualization (lead), writing – original draft (lead), writing – review and editing (lead). **Antiana C. Richardson**: methodology (support), data acquisition (support), writing – review and editing (support). **Heather H. Pua**: conceptualization (lead), methodology (lead) funding acquisition (lead), resources (lead), project administration (lead), writing – original draft (lead), writing – review and editing (lead).

## Policy on Using ChatGPT and Similar AI Tools

The authors used ChatGPT Edu (OpenAI) during manuscript preparation to assist with equation formatting, language and style editing, proofreading and iterative discussion of manuscript phrasing, titles and interpretation. The AI tool was used solely as an editorial and language‐support resource and was not used to generate original scientific concepts, synthesize scientific ideas, derive conclusions or produce experimental results or analyses. All AI‐assisted content was critically reviewed, edited, and verified by the authors, who assume full responsibility for the accuracy, integrity and originality of the work.

## Funding

This research was supported by NSF Graduate Research Fellowship Program Grant 2444112 (A.C.R.), NIH DP2 HL152426 (H.H.P.) and institutional funds from the Department of Pathology, Microbiology and Immunology at Vanderbilt University Medical Center (H.H.P.). Opinions, findings and conclusions expressed in this material are those of the authors and do not necessarily reflect the views of the funding agency.

## Ethics Statement

All mouse experiments were performed with approval from the Vanderbilt Institutional Animal Care and Use Committee. All material in this manuscript is original and has not been published elsewhere. All work in this manuscript upholds principles of rigorous and reproducible research in science.

## Conflicts of Interest

The authors declare no conflict of interest.

## Supporting information




**Supporting information**: jex270162‐sup‐0001‐TableS1.xlsx

**Supporting information**: jex270162‐sup‐0002‐TableS2.xlsx

**Supporting information**: jex270162‐sup‐0003‐TableS3.xlsx

**Supporting information**: jex270162‐sup‐0004‐TableS4.xlsx

**Supporting information**: jex270162‐sup‐0005‐TableS5.xlsx

**Supporting information**: jex270162‐sup‐0006‐TableS6.xlsx

**Supporting information**: jex270162‐sup‐0007‐TableS7.xlsx

**Supporting information**: jex270162‐sup‐0008‐TableS8.xlsx

**Supporting informaiktion**: jex270162‐sup‐0009‐TableS9.xlsx

**Supporting information**: jex270162‐sup‐0010‐TableS10.xlsx

**Supporting information**: jex270162‐sup‐0011‐FigureS1‐S6.docx

## Data Availability

RNA sequencing data is available on the Gene Expression Omnibus (GSE333855). Other data supporting the findings of this study are available from the corresponding author upon request.
