# Supplementary material for: RNY1 Is Heterogeneously Partitioned in Inflamed Airway Fluid and Modulates Pro‐Inflammatory Macrophage Transcriptional Programming
Source: J Extracell Biol. 2026 Jul 3;5(7):e70162. doi: 10.1002/jex2.70162 (PMC13329265; doi:10.1002/jex2.70162)
Supplement: Supplementary file 11 — Supporting information: jex270162‐sup‐0011‐FigureS1‐S6.docx [file JEX2-5-e70162-s006.docx]

**Supplemental Information**

**RNY1 is heterogeneously partitioned in inflamed airway fluid and modulates pro-inflammatory macrophage transcriptional programming**

Cherie E. Saffold, BS^1,2,3,4^; Antiana C. Richardson, BS^1,2,3,4^; Heather H. Pua MD, PhD^1,2,3,4*^

^1^Department of Pathology, Microbiology, and Immunology, Vanderbilt University Medical Center, Nashville, TN, USA

^2^Vanderbilt Institute for Infection, Immunology, and Inflammation, Vanderbilt University Medical Center, Nashville, TN, USA

^3^Vanderbilt Center for Immunobiology, Vanderbilt University Medical Center, Nashville, TN, USA

^4^Vanderbilt Center for Extracellular Vesicle Research, Vanderbilt University, Nashville, TN, USA

*Correspondence to: Heather H. Pua, [heather.pua@vumc.org](mailto:heather.pua@vumc.org), ORCID: 0000-0002-9271-2608, C3321A Medical Center North, 1161 21^st^ Avenue South, Nashville, TN 37232, 615-343-3912

Additional information: Cherie E Saffold ([cherie.e.saffold@vanderbilt.edu,](mailto:cherie.e.saffold@vanderbilt.edu) ORCID: 0000-0002-0347-2068), Antiana C Richardson ([antiana.c.richardson@vanderbilt.edu,](mailto:antiana.c.richardson@vanderbilt.edu) ORCID: 0000-0002-5595-8078)


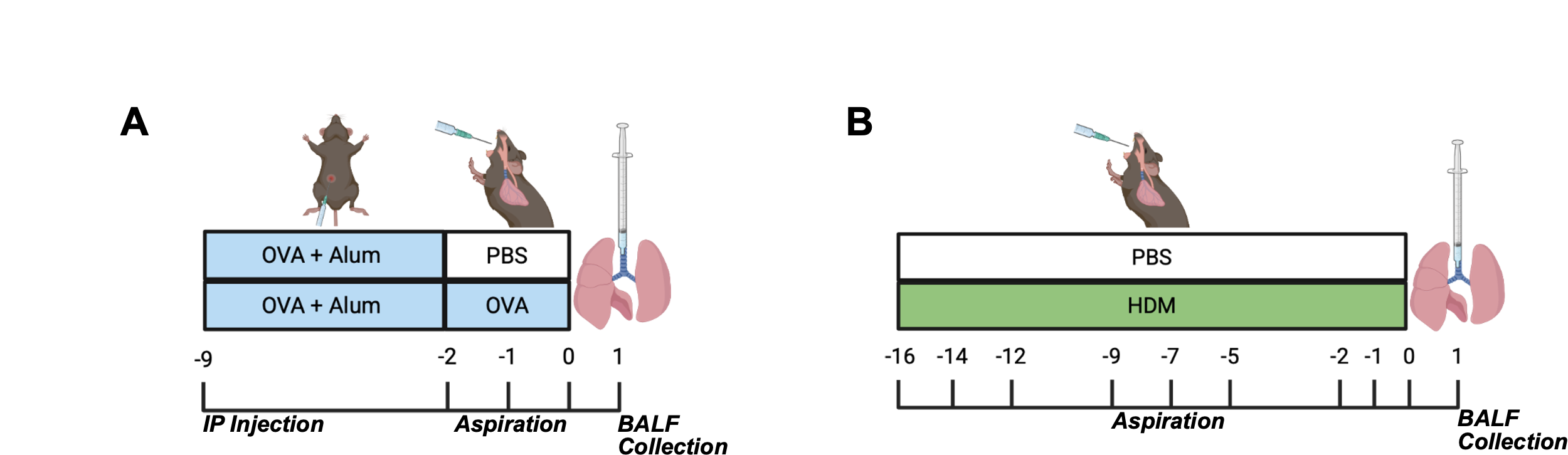


**Supplemental Figure 1:**Murine models of airway inflammation. **A and B)** Schema of OVA **(A)** and HDM **(B)** models of airway inflammation. OVA = ovalbumin, HDM = house dust mite.


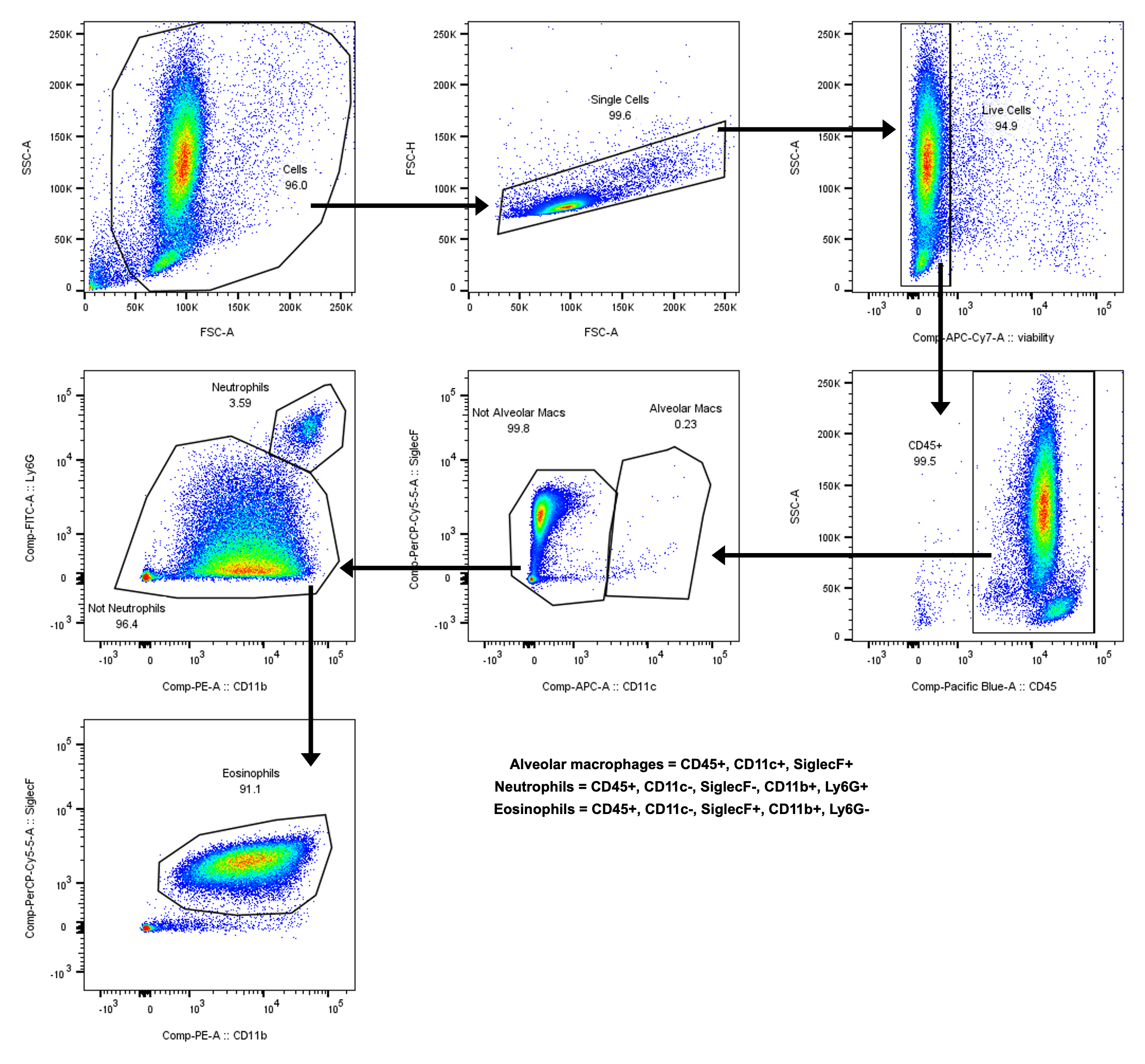


**Supplemental Figure 2:** Flow cytometry gating strategy for alveolar macrophages, neutrophils, and eosinophils in BALF.

**
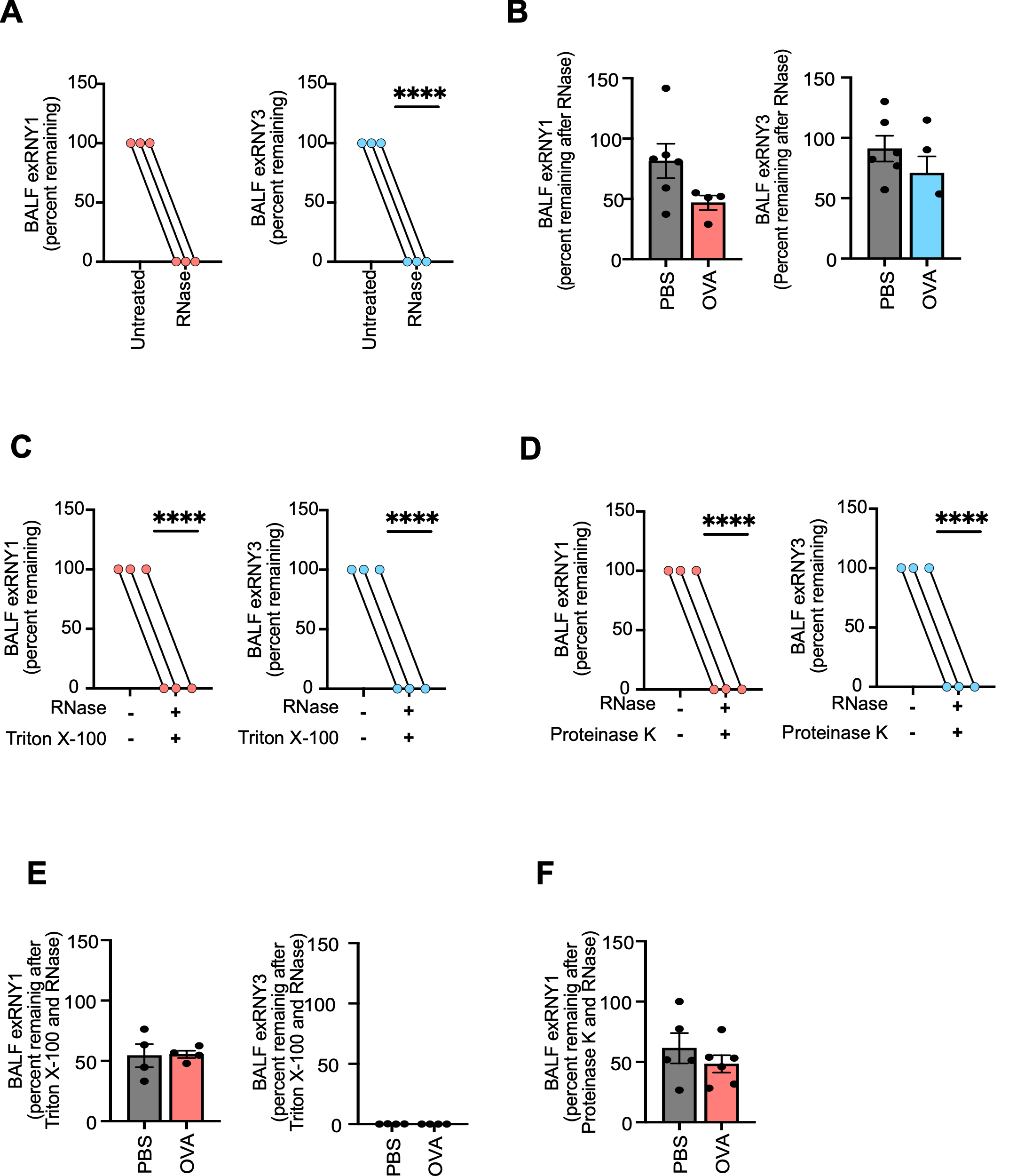
**

**Supplemental Figure 3:** **A-C:** YRNA qPCR of **(A)**, Triton X-100 + RNase- **(B)** and Proteinase K + RNase- **(C)** treated BALF RNA pre-extracted with phenol-chloroform. Data is expressed as the percentage of remaining YRNA compared to an untreated control. One sample student's t-test. N = 3 from 2 independent experiments. **D)** YRNA qPCR of RNase-treated BALF from PBS and OVA-challenged mice. Data is expressed as the percentage of remaining YRNA compared to an untreated control. Unpaired student's t-test. N = 4-6 from 2-3 independent experiments. One RNY1 value was removed as an outlier using the ROUT outlier test. **E and F)** YRNA qPCR of Proteinase K + RNase-treated **(E)** and Triton X-100 + RNase-treated **(F)** BALF from PBS and OVA-challenged mice. Data is expressed as the percentage of remaining YRNA compared to an RNase-treated control. Unpaired student's t-test. N = 4-6 from 2 independent experiments. For all panels: *= P ≤ 0.05, **= P ≤ 0.01, ***= P ≤ 0.001, ****= P ≤ 0.0001. Bars represent the mean +/- standard error of the mean.


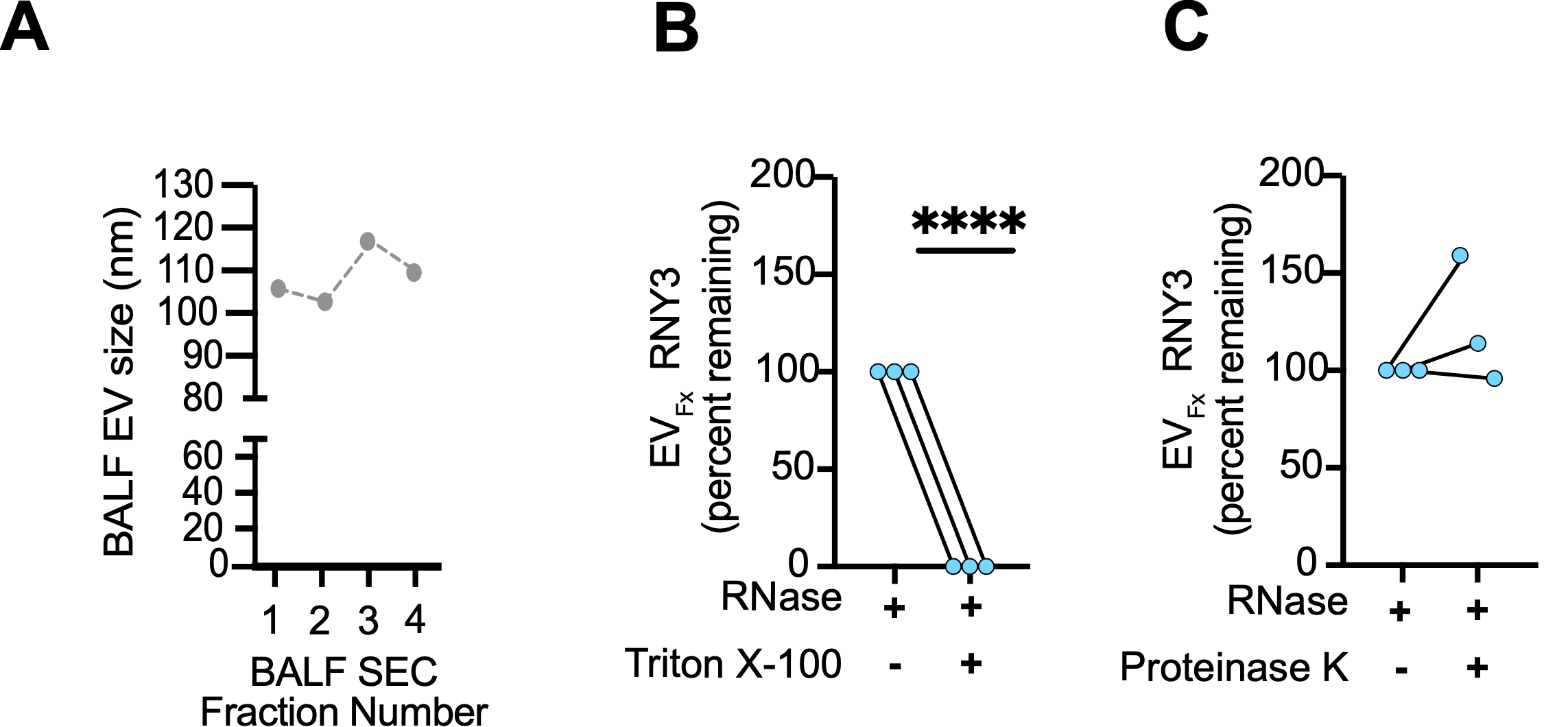


**Supplemental Figure 4: A)** Particle size quantification in BALF SEC fractions 1-4. N = 1 from 1 independent experiment. **B and C)** RNY3 qPCR of EV_Fx_ treated with Triton X-100 + RNase **(B)** or Proteinase K + RNase **(C)**. Data is expressed as the percentage of remaining YRNA compared to an RNase-only treated control. One sample students t-test. N = 5 from 2 independent experiments. For all panels: *= P ≤ 0.05, **= P ≤ 0.01, ***= P ≤ 0.001, ****= P ≤ 0.0001. BALF = bronchoalveolar lavage fluid, EV_Fx_ = EV-enriched fraction, Protein_Fx_ = protein-enriched fraction.

**
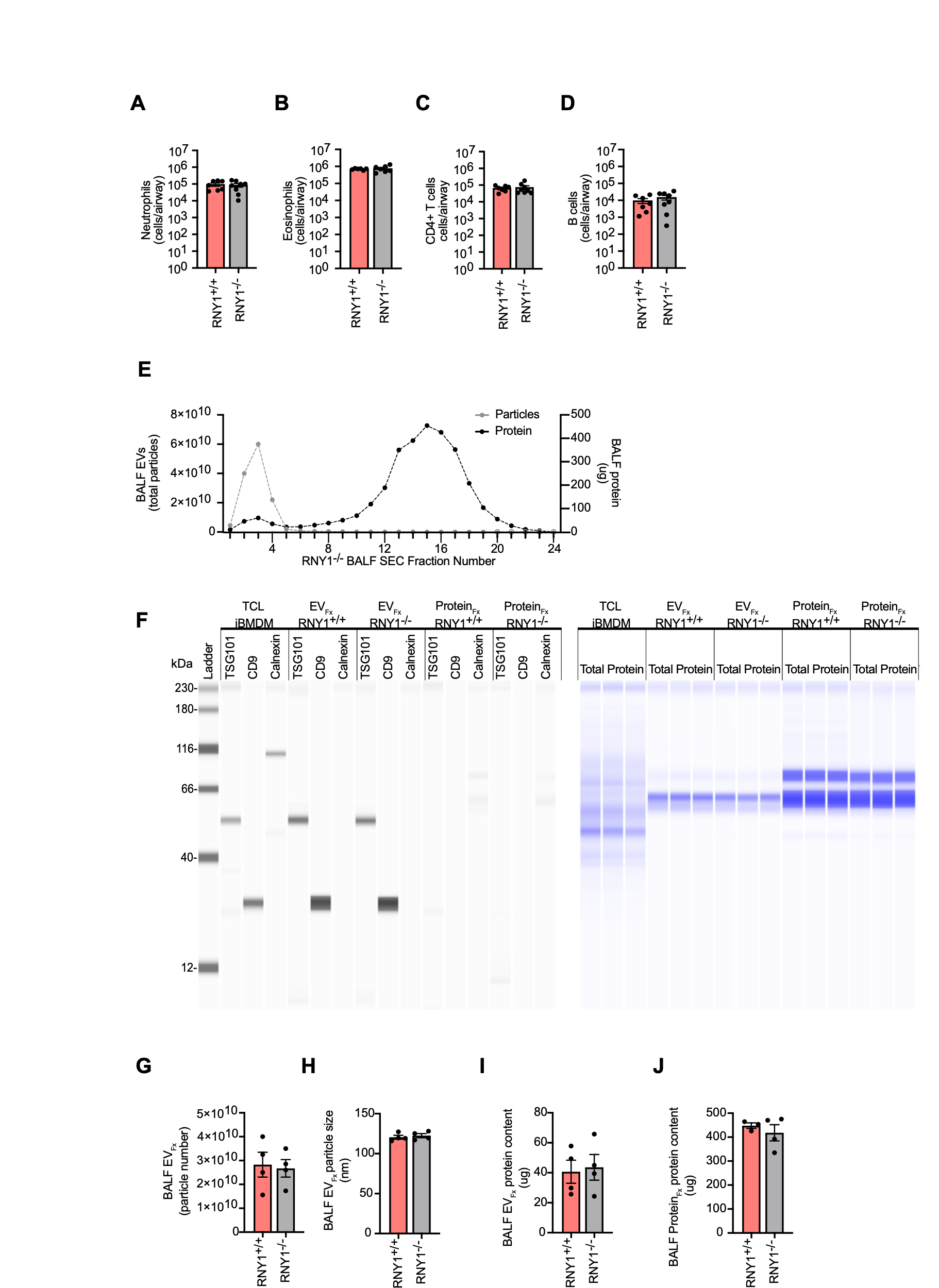
**

**Supplemental Figure 5:** **A-D)** BALF neutrophil **(A)**, eosinophil **(B)**, CD4^+^ T cell **(C)**, and B cell **(D)** counts from OVA-challenged RNY1^+/+^ and RNY1^-/-^ mice determined via flow cytometry. Cell counts and flow cytometry data were obtained 1 day after the last OVA challenge. Welch's t-test. N = 6-8 from 2 independent experiments. **E)** Particle and protein quantification in RNY1^-/-^ BALF SEC fractions. **F)** Immortalized BMDMs were used as a technical control for calnexin detection **G-I)** BALF particle counts **(G)**, size **(H)**, and protein content **(I)** in RNY1^+/+^ and RNY1^-/-^ EV_Fx_. **J)** Protein quantification of RNY1^+/+^ and RNY1^-/-^  Protein_Fx_. For all panels: *= P ≤ 0.05, **= P ≤ 0.01, ***= P ≤ 0.001, ****= P ≤ 0.0001. Bars represent the mean +/- standard error of the mean. Open circles indicate no detection. BALF = bronchoalveolar lavage fluid, EV_Fx_ = EV-enriched fraction, Protein_Fx_ = protein-enriched fraction, BMDM = bone marrow-derived macrophage.

**
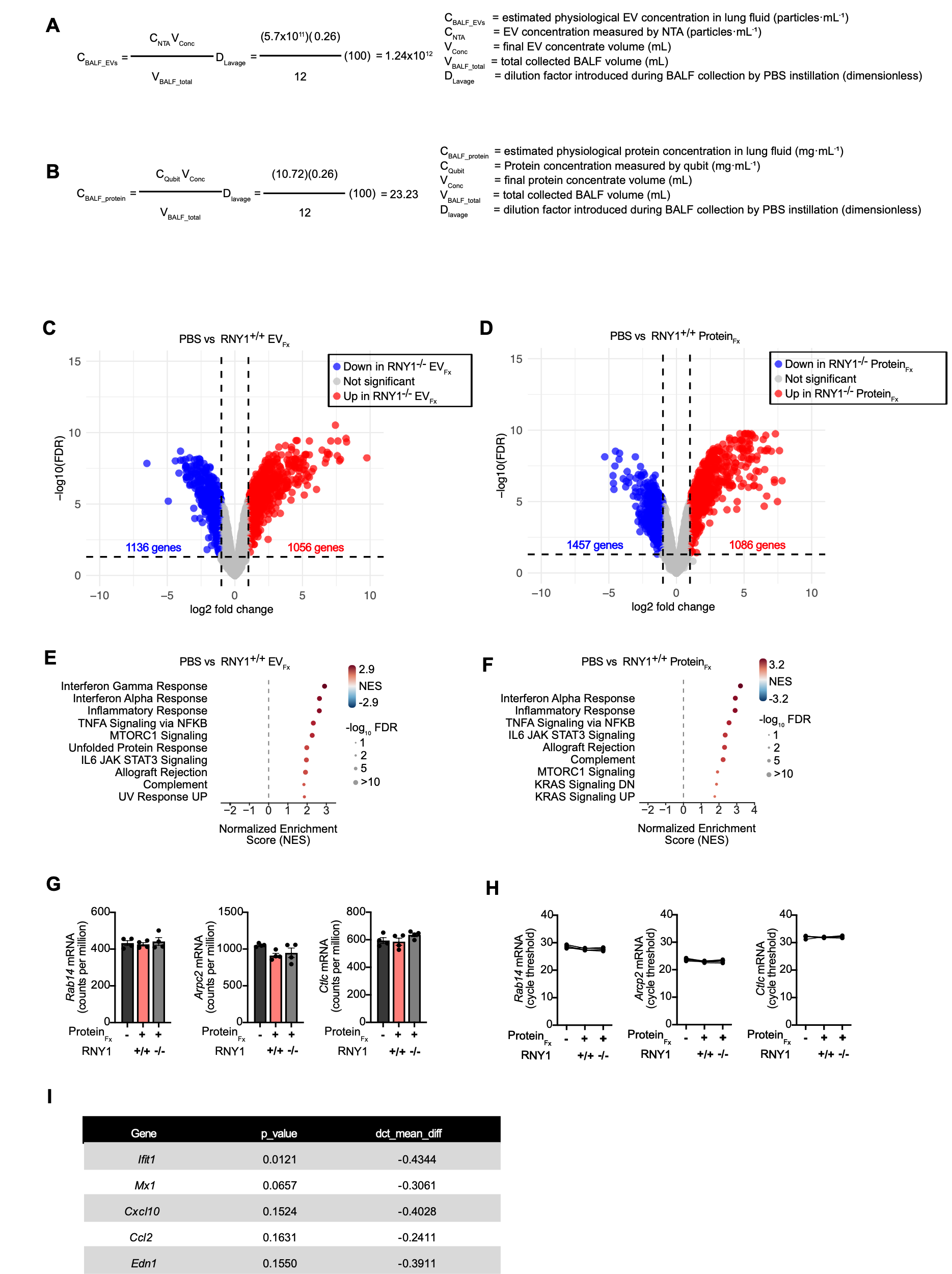
**

**Supplemental Figure 6:** **A and B)** Equations used to approximate physiological concentrations of BALF EV **(A)** and protein **(B). C and D)** Volcano plots of DEGs in BMDMs treated with EV_Fx_ **(C)** and Protein_Fx_ **(D)** compared to the untreated control. Genes with CPM <3 were removed from analysis. Significance cutoffs are FDR > 0.05 and log2 Fold Change > 1. **E and F)** Top 10 pathways identified in gene set enrichment analysis using the Hallmark gene set. Dots represent the -log_10_ FDR and NES of pathways in EV_Fx_ **(E)** or Protein_Fx_ **(F)** treated BMDMs compared to the PBS-treated control. **G)** Candidate housekeeping gene read counts per million. **H)** Candidate housekeeping gene cycle threshold values. The average cycle threshold value for all three genes was used to calculate ΔCt values for genes analyzed in Fig. 5J. **Q)** P-values and mean differences between RNY1^+/+^ Protein_Fx_-, and RNY1^-/-^ Protein_Fx_-treated BMDM candidate gene −ΔCt values. For all panels: *= P ≤ 0.05, **= P ≤ 0.01, ***= P ≤ 0.001, ****= P ≤ 0.0001. Bars represent the mean +/- standard error of the mean. Open circles indicate no detection. EV_Fx_ = EV-enriched fraction, Protein_Fx_ = protein-enriched fraction, BMDM = bone marrow-derived macrophage.
